# Sleep duration and satisfaction among physicians in tertiary public hospitals in China: a large sample national survey

**DOI:** 10.1186/s12995-021-00298-z

**Published:** 2021-03-05

**Authors:** Yinuo Wu, Feng Jiang, Shichao Wu, Yuanli Liu, Yi-Lang Tang

**Affiliations:** 1 Chinese Academy of Medical Sciences & Peking Union Medical College, Beijing, China; 2 Institute of Health Yangtze River Delta, Shanghai Jiao Tong University, 1954 Huashan, Xuhui District, Shanghai, 200030 China; 3 Peking University Shenzhen Hospital, Shenzhen, China; 4 School of Health Policy and Management, Chinese Academy of Medical Sciences & Peking Union Medical College, 5 Dongdan Santiao, Dongcheng District, Beijing, 100730 China; 5 Department of Psychiatry and Behavioral Sciences, Emory University, Atlanta, GA USA; 6 Atlanta VA Medical Center, Decatur, GA USA

**Keywords:** Short sleep duration, Prevalence, Physicians, Tertiary public hospitals, China

## Abstract

**Objectives:**

To investigate the sleep duration and level of satisfaction among physicians in tertiary public hospitals in China, and to explore associated factors.

**Methods:**

A national online cross-sectional survey was conducted. Totally 20,786 physicians from 136 hospitals participated in the survey. Data were collected using an online self-reported questionnaire. Descriptive and logistic regression statistics were performed using the STATA software.

**Results:**

The mean total sleep duration was 6.37 ± 0.87 h. Of all participants, 61.06% (*n* = 12,691) reported short sleep duration (less than 7 h per day). 46.97% (*n* = 9764) were not satisfied with their sleep. An older age and job-related factors (longer working hours per week, specialty including internal medicine, Ob/GYN and emergency medicine, working more night shifts, heavier workload, and working in East China) were significantly associated with reported short sleep duration.

**Conclusions:**

The majority of physicians in Chinese public hospitals experienced insufficient sleep duration. Changes are required to improve the wellbeing of physicians and patient outcomes.

## Introduction

Sufficient sleep is crucial to human health, and it has been recommended that adults aged 18–60 years should have no less than 7 h of sleep per night to maintain optimal health [1]. Short sleep duration (defined as less than 7 h of sleep per night) has been linked to multiple adverse health outcomes [2]. Meanwhile, sleep insufficiency also impairs people’s cognitive performance, and increases the risk of performance errors, such as transportation accidents [3].

In general, physicians with short sleep durations are more likely to have poor health conditions, increased alcohol and medication use, and serious medical errors [4, 5]. Therefore, it is important to allow sufficient sleep in physicians to ensure their wellbeing and patients’ wellbeing and safety.

The reported sleep duration and satisfaction in physicians vary widely in different countries and different samples. According to the National Health Interview Survey (NHIS) in the US, which consisted of a series of cross-sectional national surveys, the prevalence of short sleep duration (less than 7 h/night) was between 29 and 31% in physicians of different specialties [6]. In the survey of Behavioral Risk Factor Surveillance System (BRFSS), 39.7% of American healthcare practitioners slept less than 7 h per day [7]. In Japan, Wada et al. surveyed 3369 physicians by mail and found that 87.23% of them slept less than 7 h for days not working overnight [8].

To the best knowledge of the authors, there have been no national surveys focusing on this topic in China. According to the China Sub-optimal Health Survey (CSHS), which included 18,631 participants, the average sleep duration for professionals was 7.87 ± 1.08 h per day [9].

Our study was set to collect data on sleep duration and satisfaction of physicians working in tertiary public hospitals in China. We also collected demographic and work-related data and examined the associations between sleep, especially short sleep duration.

## Materials and methods

### Study design and samples

The study was part of China’s Healthcare Improvement Initiative Survey in 2017, which was sponsored by the National Health Commission of China. The objectives of the survey were to improve healthcare quality and satisfaction of patients and medical staff in tertiary public hospitals. The study was conducted on December 18–31, 2017. The study recruited 136 tertiary public hospitals across 31 provinces in mainland China [10]. These hospitals accounted for 6.45% of all the tertiary hospitals, delivered 12.28% of the inpatient care among tertiary hospitals, 10.90% of all physicians in tertiary hospitals [11]. In the Chinese healthcare system, tertiary hospitals play a critical role. Based on their employee’s ID codes on the hospital staff lists, physicians were sampled through a systematic sampling method in each participating hospital. We invited 167 physicians from each hospital to participate. The project was conducted anonymously through WeChat, a widely used social media application in mainland China.

### Measures

Sleep duration was evaluated using a self-report item. “How many hours do you sleep every night on average?” We also assessed the satisfaction on sleep duration (1 = very dissatisfied, 3 = neutral, 5 = very satisfied) and workload (1 = very mild, 3 = neutral, 5 = very heavy), with a 5-point Likert scale. The Cronbach’s Alpha Coefficient was 0.73 for these items.

Additionally, we collected participants’ sociodemographic data, including age, sex, marital status, the number of children, educational level, as well as job-related factors, including their department, having an administration position or not, the frequency of night shift, working days and working hours per week, hospital type and regions of the hospital.

### Statistical analysis

Descriptive analyses were conducted for the variables. The item of sleep hours was a continuous variable. According to the cut-off point, 7 h per day, physicians were divided into two groups, short sleep duration group or others.

We used the multiple logistic regression to explore the relationship between short sleep duration and related factors. Stata 15 (StataCorpLP, College Station, TX, USA) was used for these statistical analyses. All of the tests were two-sided, and the statistical significance was defined as *p* < 0.05.

## Results

### Description of sample characteristics and related factors

After removing 974 participants with incomplete data, 20,786 physicians were included in the analysis. The response rate was 95.52%. Their sociodemographic characteristics were shown in Table 1.

**Table 1:** Characteristics of participants (*N* = 20,786)

	Frequency	Percentage (%)
Sex		
Male	9056	43.57
Female	11,730	56.43
Age (year)		
< =29	3711	17.85
30–39	10,383	49.95
40–49	4633	22.29
> =50	2059	9.91
Marital status		
Not married	3643	17.53
Married	16,658	80.14
Divorced or separated	485	2.33
Children		
None	6085	29.27
One child	12,162	58.51
More than one	2539	12.21
Educational level^a^		
Bachelor of Mecicine	5952	28.64
Additional Master’s or doctorate degree	14,834	71.37
Department		
General medicine	4878	23.47
General surgery	3151	15.16
Gynecology and obstetrics	2940	14.14
General pediatrics	1762	8.48
Emergency medicine	696	3.35
Internal medicine subspecialty^b^	3756	18.07
General surgery subspecialty^c^	3603	17.33
Administration position		
Yes	2568	12.35
No	18,218	87.65
Night shift per month		
0–4 times	11,812	56.83
> 4 times	8974	43.17
Hospital type		
General	8754	42.11
Traditional Chinese Medicine	5214	25.08
Other special types^d^	6818	32.80
Region		
East China	8028	38.62
Middle China	5385	25.91
West China	7373	35.47
Workload		
Very heavy/Heavy	12,794	61.55
Moderate	6611	31.80
Very mild/Mild	1381	6.64

^a^In China, medical school graduates are awarded with a bachelor degree of medicine (similar to European and Russian system). Some obtained an master’s or doctorate degree in addition to their medical degree

^b^Including Traditional Chinese Medicine department, rehabilitation department, geriatrics department, etc.

^c^Including reproductive department, plastic surgery department, ophthalmology department, etc.

^d^Mainly Maternal and Child Health Hospitals

Most physicians reported that the workload was heavy or very heavy. They reported to have worked 5.76 ± 0.71 days and 51.05 ± 19.36 h per week, and 69.60% of them worked longer than 44 h per week, the limit set by the Labor Law of China [12].

### Description of sleep duration and satisfaction

The average sleep duration of physicians was 6.37 ± 0.87 h per day. 61.06% of physicians reported that they slept less than 7 h per day, or short sleep duration. 11.87% reported less than 6 h of sleep and 1.27% reported less than 5 h of sleep.

46.97% of physicians were dissatisfied or very dissatisfied with their sleep duration. Among the physicians with short sleep duration, 63.32% of them were dissatisfied or very dissatisfied with their sleep duration.

### Logistic regression analysis of short sleep duration and associated factors

The multivariate logistic regression model showed that both age and job-related factors (working hours per week, department, number of night shifts, workload, and region) were significantly associated with short sleep duration (see Table 2).

**Table 2:** Logistic regression analysis of short sleep duration

	Odds Ratio	95.0% CI (Lower)	95.0% CI (Upper)
Male (ref. female)	0.95	0.89	1.09
Age (ref. ≤ 29)			
30–39	1.31**	1.19	1.44
40–49	1.65**	1.47	1.86
≥ 50	1.90**	1.65	2.20
Marital status (ref. not married)			
Married	0.94	0.84	1.05
Divorced or separated	1.18	0.95	1.48
Children (ref. None)			
One	0.99	0.89	1.09
More than one	1.06	0.94	1.20
Educational level (ref. Bachelor’s degree)	1.03	0.96	1.10
Working hours per week	1.01**	1.01	1.01
Department (ref. General surgery subspecialty)			
General medicine	1.22*	1.10	1.34
General surgery	1.10	0.98	1.23
Gynecology and obstetrics	1.12*	1.01	1.25
General pediatrics	1.06	0.93	1.20
Emergency medicine	1.21*	1.01	1.46
Internal medicine subspecialty	0.99	0.89	1.09
Administration position (ref. No)	1.00	0.90	1.10
Night shift per month (ref. 0–4 times)	1.31**	1.23	1.40
Workload (ref. Mild level)			
Moderate	1.83**	1.62	2.08
Heavy level	3.73**	3.30	4.22
Hospital type (ref. General)			
Traditional Chinese Medicine	1.02	0.95	1.10
Other special types	1.04	0.96	1.12
Region (ref. West China)			
East China	1.14**	1.07	1.23
Middle China	0.92*	0.85	0.99

*p < 0.05, **p < 0.001

Using the age group of age 29 or younger as a reference, older age groups were more likely to report a short sleep duration. Specifically, OR = 1.31 in 30–39 years group (*p <* 0.001), OR = 1.65 in 40–49 years group(*p <* 0.001), and OR = 1.90 in ≥50 years group (*p <* 0.001) respectively.

Longer working hours were also significantly associated with short sleep duration. One hour increase each week was associated with 1% increase in the likelihood to have a short sleep duration in physicians (OR = 1.01, *p <* 0.001). Compared to general surgery subspecialty department, physicians in general medicine department, OB/GYN, emergency medicine department, were all more likely to have short sleep duration (OR = 1.22, *p <* 0.05; OR = 1.12, *p <* 0.05; OR = 1.21, *p <* 0.05; respectively). Comparing with those working 0–4 night shifts per month, physicians who had more than 4 night shifts every month were more likely to have short sleep duration (OR = 1.31, *p <* 0.001). Physicians reported to have moderate workload or heavy/very heavy workload, were significantly to have short sleep duration, when compared to mild/very mild workload (OR = 1.83, *p <* 0.001; OR = 3.73, *p <* 0.001; respectively). Meanwhile, physicians in East China were more likely to have a short sleep duration than those in west China (OR = 1.14, *p <* 0.001); while physicians in Central China were less likely to have a short sleep duration than these in west China (OR = 0.92, *p <* 0.05).

## Discussion

To our best knowledge, this study was the first one to report sleep duration, level of satisfaction, and short sleep duration prevalence in Chinese physicians within a large national sample. We found that more than 60% of physicians reported they slept less than 7 h per day. Meanwhile, nearly half of physicians were not satisfied with their sleep duration. These findings are concerning and of importance to hospital administrators and policy makers. Furthermore, we found age and some job-related factors were associated with short sleep duration, which was in line with previous findings [13].

The high prevalence of insufficient sleep in Chinese physicians was similar in other Asian countries. For example, 87.23% of Japanese physicians slept less than 7 h per day [8]. Meanwhile, only 29–39.7% of American physicians slept less than 7 h per day [6, 7]. This gap might be partly due to the differences in workload, as the workload was a significant influence factor for sleep duration in physicians [14]. In this survey, more than half of the participants reported a high workload. According to another study, the workload of Chinese physicians increased dramatically from 1998 to 2016 [15]. At the same time, in Asian countries, the extra work hours in hospitals generally were not paid, so hospitals were motivated to add more workload [16]. As a result, Asian physicians have to endure heavy workload and long work hours, and often at the expense of their sleep. Comparatively, in recent decades, the working hours of all American physicians have steadily decreased [17].

A few limitations of this study need to be acknowledged here. First, this was a cross-sectional study, which can not reveal the cause of the short sleep duration. Second, we used self-reporting data of sleep duration, instead of sleep diary recordings. And we did not study their quality of sleep. Therefore recall bias cannot be ruled out. Third, the sample was from tertiary public hospitals, so the findings may not be generalizable to all physicians in China, especially those working in rural areas.

In summary, the prevalence of short sleep duration in Chinese physicians was high and nearly half participants were not satisfied with their sleep. Hospital administrators and policymakers need to be aware of this important issue and changes are required to improve the wellbeing of physicians and patient outcomes.

## Data Availability

The datasets analyzed during the current study are available from the corresponding author on reasonable request.

## References

[1] Consensus Conference P, Watson NF, Badr MS, Belenky G, Bliwise DL, Buxton OM, Buysse D, Dinges DF, Gangwisch J, Grandner MA (2015). Joint Consensus statement of the American Academy of sleep medicine and Sleep Research Society on the recommended amount of sleep for a healthy adult: methodology and discussion. Sleep.

[2] Nakata A (2011). Work hours, sleep sufficiency, and prevalence of depression among full-time employees: a community-based cross-sectional study. J Clin Psychiatr.

[3] Garbarino S, Magnavita N, Guglielmi O, Maestri M, Dini G, Bersi FM, Toletone A, Chiorri C, Durando P (2017). Insomnia is associated with road accidents. Further evidence from a study on truck drivers. PLoS One.

[4] Kalmbach DA, Arnedt JT, Song PX, Guille C, Sen S. Sleep Disturbance and Short Sleep as Risk Factors for Depression and Perceived Medical Errors in First-Year Residents. Sleep. 2017;40(3):73–80. 10.1093/sleep/zsw073 PMC6084763 28369654

[5] Mansukhani MP, Kolla BP, Surani S, Varon J, Ramar K (2012). Sleep deprivation in resident physicians, work hour limitations, and related outcomes: a systematic review of the literature. Postgrad Med.

[6] Jackson CL, Kawachi I, Redline S, Juon HS, Hu FB (2014). Asian-white disparities in short sleep duration by industry of employment and occupation in the US: a cross-sectional study. BMC Public Health.

[7] Shockey TM, Wheaton AG (2017). Short sleep duration by occupation group - 29 states, 2013-2014. MMWR Morb Mortal Wkly Rep.

[8] Wada K, Yoshikawa T, Goto T, Hirai A, Matsushima E, Nakashima Y, Akaho R, Kido M, Hosaka T (2010). National survey of the association of depressive symptoms with the number of off duty and on-call, and sleep hours among physicians working in Japanese hospitals: a cross sectional study. BMC Public Health.

[9] Sun W, Yu Y, Yuan J, Li C, Liu T, Lin D, Lau A, Zhong C, Xu T, Shan G (2015). Sleep duration and quality among different occupations--China national study. PLoS One.

[10] Zhou H, Han X, Zhang J, Sun J, Hu L, Hu G, Wu S, Zhao P, Jiang F, Liu Y (2018). Job satisfaction and associated factors among medical staff in tertiary public hospitals: results from a National Cross-Sectional Survey in China. Int J Environ Res Public Health.

[11] National Health Commission of the People's Republic of China (2019). China health statistical yearbook.

[12] China Legal System Publishing House (2018). Labor Law of the People’s Republic of China.

[13] Luckhaupt SE, Tak S, Calvert GM (2010). The prevalence of short sleep duration by industry and occupation in the National Health Interview Survey. Sleep.

[14] Tucker P, Bejerot E, Kecklund G, Aronsson G, Akerstedt T (2015). The impact of work time control on physicians' sleep and well-being. Appl Ergon.

[15] Fu Y, Schwebel DC, Hu G. Physicians' Workloads in China: 1998-2016. Int J Environ Res Public Health. 2018;15(8):1649–53. 10.3390/ijerph15081649 PMC6121441 30081497

[16] Ehara A (2013). Labor law violations in Japanese public hospitals from march 2002 to march 2011. Pediatr Int.

[17] Staiger DO, Auerbach DI, Buerhaus PI (2010). Trends in the work hours of physicians in the United States. Jama.

